# Health Perceptions and Misconceptions Regarding COVID-19 in China: Online Survey Study

**DOI:** 10.2196/21099

**Published:** 2020-11-02

**Authors:** Jiawei Zhou, Bishwajit Ghose, Ruoxi Wang, Ruijun Wu, Zhifei Li, Rui Huang, Da Feng, Zhanchun Feng, Shangfeng Tang

**Affiliations:** 1 School of Medicine and Health Management Tongji Medical College Huazhong University of Science and Technology Wuhan China; 2 School of Pharmacy Tongji Medical College Huazhong University of Science and Technology Wuhan China; 3 China National Center for Biotechnology Development Beijng China

**Keywords:** COVID-19, perceptions, knowledge, coronavirus, SARS-CoV-2, pandemic, rapid, online, surveys, public health

## Abstract

**Background:**

Great efforts have been made to prevent the spread of COVID-19, including national initiatives to promote the change of personal behaviors. The lessons learned from the 2003 SARS outbreak indicate that knowledge and attitudes about infectious diseases are related to panic among the population, which may further complicate efforts to prevent the spread of infectious diseases. Misunderstandings may result in behaviors such as underestimation, panic, and taking ineffective measures to avoid infection; these behaviors are likely to cause the epidemic to spread further.

**Objective:**

The purpose of this study is to assess public health perceptions and misunderstandings about COVID-19 in China, and to propose targeted response measures based on the findings to control the development of the epidemic.

**Methods:**

The study was conducted in April 2020 through an online survey, with participants in 8 provinces in Eastern, Central, and Western China. We designed a questionnaire with a health knowledge section consisting of 5 questions (4 conventional questions and 1 misleading question) on clinical features of and preventive measures against COVID-19. Descriptive statistics, chi-square analysis, binary logistic regression, and Mantel-Haenszel hierarchical analysis were used for statistical analysis.

**Results:**

In total, 4788 participants completed the survey and the mean knowledge score was 4.63 (SD 0.67), gained mainly through experts (76.1%), television (60.0%), newspapers (57.9%), and opinions (46.6%) and videos (42.9%) from social media. Compared to those who obtained information from only 1 or 2 channels, people who obtained information from >3 channels had increased health perception and a better ability to identify misleading information. Suggestions from experts were the most positive information source (χ2=41.61), while information on social media was the most misleading. Those aged >60 years (OR 1.52, 95% CI 1.10-2.11), those with a lower or middle income (OR 1.36, 95% CI 1.00-1.83), those not working and not able to work (OR 1.83, 95% CI 1.04-3.21), those with a household income <100,000 RMB (<US $14,954; OR 1.34, 95% CI 1.08-1.67), and those with >2 suspected symptoms (OR 2.95, 95% CI 1.50-5.80) were more likely to be misled by videos on social media, but the error correction effect of expert advice was limited in these groups.

**Conclusions:**

Multiple information channels can improve public health perception and the identification of misleading information during the COVID-19 pandemic. Videos on social media increased the risk of rumor propagation among vulnerable groups. We suggest the government should strengthen social media regulation and increase experts’ influence on the targeted vulnerable populations to reduce the risk of rumors spreading.

## Introduction

In December 2019, the novel coronavirus SARS-CoV-2 caused an outbreak of COVID-19 in Wuhan, China [1,2]. COVID-19 is spread by human-to-human transmission via droplets or direct contact [3-5], and the clinical symptoms of COVID-19 include fever, cough, fatigue, and gastrointestinal infection symptoms [6-8]. Major transmission hotspots were brought under control in China, but others subsequently sprouted worldwide. Since late February 2020, the daily number of new cases has been higher in other regions of the world [9]. On March 11, 2020, the World Health Organization (WHO) declared COVID-19 a pandemic [10], and by May 17, 2020, there were more than 4,500,000 confirmed cases and over 300,000 reported deaths from COVID-19 worldwide [11]. The COVID-19 pandemic has clearly entered a new stage, with rapid spread in countries outside China. All members of society have to understand and practice measures for self-protection and the prevention of transmission [12].

Individual behavior is crucial for controlling the transmission of COVID-19. Although there have been satisfactory results regarding the safety, tolerability, and immunogenicity of a COVID-19 vaccine [13], a vaccine still faces many failure risks, and producing and administering vaccines to millions of people worldwide takes time. In China, the epidemic situation was brought under control by the implementation of multifaceted public health measures including but not limited to intensive intracity and intercity traffic restriction, social distancing measures, home confinement and centralized quarantine, and improvement of medical resources [5,14,15]. In Western democracies, personal action, rather than government action, might be the most important issue. Early self-isolation, seeking medical advice remotely unless symptoms are severe, and social distancing are key [5]. Strong infection control measures are the primary intervention for minimizing the spread of the virus in both health care settings and the community [16,17]. According to the Knowledge-Attitude-Belief (KAP) theory, knowledge is the basis of behavior change, and belief and attitude are the driving forces of behavior change. Therefore, public perception of dealing with highly infectious respiratory diseases plays a vital role in limiting the spread of the infection [18,19].

The lessons learned from the 2003 SARS outbreak suggest that knowledge and attitudes about infectious diseases are associated with the level of panic emotion among the population, which could further complicate efforts to prevent the spread of a disease [20,21]. Behaviors like underestimation, panic, and taking ineffective measures to avoid infection may affect the fight against COVID-19 [22]. Therefore, understanding what the general public knows regarding COVID-19 and which misperceptions they hold about the condition is important for public health authorities in China and other countries, who aim to design effective information campaigns for epidemic prevention and control [23]. For this study, we conducted a rapid online survey in April 2020 to investigate health perceptions and misperceptions regarding COVID-19 among the general population in China and to identify vulnerable populations and channels being used to spread misinformation.

## Methods

### Study Design

This study used directional stratified convenient sampling to select residents in Eastern, Central, and Western China. To ensure sufficient representativeness, we included not only provinces with severe epidemics, but also provinces with relatively mild epidemics. Therefore, two provinces with severe epidemics and one province with fewer epidemics were selected in the Eastern, Central, and Western regions at the time of sampling. In April 2020, as the overall epidemic situations in the central and eastern regions were relatively severe, we defined severe epidemics as >1000 cases and less severe epidemics as <500 cases. In addition, since the overall epidemic situation in the western region was relatively less serious, provinces with >500 cases were regarded as relatively severe, and provinces with <200 cases were regarded as relatively mild. Hubei, Hunan, and Shanxi provinces were selected in Central China. In Eastern China, Guangdong, Zhejiang, and Fujian provinces were selected. Due to similar local conditions and customs in Sichuan and Chongqing, we only selected one of the two provinces with relatively higher prevalence of COVID-19 in Western China; Sichuan and Gansu provinces were included. Based on cities’ populations and economic influence in the provinces, the provincial capital city and another city were purposely selected for each province. In each city, both urban and rural households were selected, for a total of 60 households. All household residents aged ≥10 years were included in this survey. In total, 7118 residents from 1920 households in 8 provinces (16 cities) were included in this investigation. Due to the lower responsiveness of residents in Guangdong (Guangzhou and the Pearl River delta) and Zhejiang provinces (Hangzhou and Jinhua)—only half of the expected households completed the survey—we combined the two provinces for analysis.

According to the COVID-19 clinical and community management guidelines issued by the national health commission of the People’s Republic of China [24,25], we designed a questionnaire to determine residents’ health perceptions regarding COVID-19.

We designed a self-administered questionnaire containing 168 questions based on literature research and past experience, including questions about demographic information, physical condition, nutrition and prevention behaviors, knowledge of drug preparation and response, and knowledge level of COVID-19, personal health and risk protection, and psychological stress (Cronbach a=.782). There were 5 questions in the questionnaire designed to examine the participants’ understanding of COVID-19:

1. Washing your hands and wearing a mask frequently could help prevent COVID-19.
2. When people with COVID-19 sneeze or cough around you, is it easier to be infected?
3. Eating a lot of garlic could help prevent COVID-19.
4. Improving your immunity could help fight COVID-19.
5. If infected, older adults have the highest risk of mortality.

The above list consists of four measures to prevent COVID-19 (Q1, Q2, Q4, and Q5) and one misleading measure (Q3), all of which were answered on a right/wrong basis. Each correct answer counts for 1 point, while incorrect answers get no points. The total health perception score ranged from 0 to 5; the higher the score, the higher the knowledge level of COVID-19. Based on the above questions, we defined participants who correctly answered 3 or more of the 5 questions as “perception level above average,” and calculated the proportion of participants who answered incorrectly on the misleading question (Q3) to get the “misled” rate.

### Data Collection

This investigation was carried out from April 4 to 15, 2020. One project manager in each province was recruited to coordinate the provincial survey and organize the investigation training, and 6 local investigators were recruited based on the household income for each city to send an online questionnaire and control the investigation quality. Half of them were from rural areas and the majority were undergraduates. After receiving data collection training online, every investigator was required to directly send the online questionnaire to 20 local families in their social network, including relatives, friends, and classmates. Every eligible participant in the family was invited to fill out the online questionnaire, which they completed within an average of 15 minutes. One secret gift was sent to encourage the participants to complete the questionnaire. Due to the constraints of their own age and educational level, some older adults are unable to participate in online surveys. For this group of people, we recommended that relatives living with them obtain their answers through oral inquiry and fill out the survey according to their choices.

If it was difficult to survey 20 families connected to the investigators, a supplementary survey was conducted by other investigators to complete the household investigation.

During data collection, we used the following quality control measures:

1. We first conducted a preliminary survey to improve our questionnaire, and then grouped the investigators and trained them on how to perform the investigation.
2. Every investigator was independent.
3. Before sending the online questionnaire, the 20 households and eligible family members were asked to generate a unique questionnaire number. Those aged >60 years accounted for >15% of the participants.
4. Every family's questionnaire was sent one by one by the investigators, who delivered a message that “the one who fills the questionnaire carefully, they will receive one secret gift.” Trap questions were included to identify those who did not answer questions carefully.
5. The project manager checked the quality of every questionnaire (based on the survey time threshold value of >450 seconds) as well as the consistency of responses to two group questions.

The protocol was reviewed and ethical approval was granted by the Ethics Committee of Tongji Medical College, Huazhong University of Science and Technology (#2020S107). Oral informed consent was obtained from each participant during the online investigation. A total of 6253 residents aged ≥10 years completed the investigation, and 4788 of them were eligible. The response rate was 87.85% (6253/7118), and the valid response rate was 67.13% (4778/7118).

### Statistical Analysis

Descriptive statistical methods were used to summarize data on sociodemographic characteristics and responses to questions concerning knowledge, perceptions, and attitudes toward COVID-19. Data were summarized as frequencies (n) and percentages (%) for categorical variables. The differences in health perception among different groups and the guiding effect of different information sources on people's health perceptions were tested using the chi-square test. Binary logistic regression analyses were employed to identify the potential factors and vulnerable populations related to COVID-19 knowledge. The demographic variables and information sources were set as independent variables, and health perception and the “a lot of garlic helps prevent COVID-19” question were respectively set as the outcome variables. To further identify vulnerable groups (ie, those with a high risk of being misled by the main sources of rumor propagation), a Mantel-Haenszel hierarchical analysis of misleading factors was conducted across subpopulations. Unstandardized regression coefficients (β) and odds ratios (ORs) and their 95% CIs were used to quantify the associations between variables and misconceptions regarding COVID-19. Data analyses were performed using Statistical Package for the Social Sciences (SPSS) software (Version 19.0; IBM Corp). *P*<.05 was considered statistically significant.

## Results

### Sample Characteristics

The average age of the 4788 participants was 40.9 years (SD 18.51 years, range 10-93 years), 53.0% (n=2540) of them were women, and older adults (>60 years) accounted for 17.0% (n=814) of the total sample. Furthermore, 1155 (24.1%) participants were current students and half of them had a bachelor’s degree or higher. In addition, 2191 (45.8%) participants were from Central China, 64.0% (n=3065) of them lived in urban areas, and only 8.7% (n=418) of participants lived alone during the COVID-19 epidemic. Detailed demographic characteristics data are shown in Table 1.

**Table 1:** Demographic characteristics of participants and knowledge level of COVID-19 by demographic variables.

Characteristics		Participants, n (%)	Perception above average level, n (%)	Misled rates, n (%)
**Age (years)**			*P*<.001	*P*<.001
	≤20	599 (12.5)	563 (94.0)	65 (10.9)
	21-40	1774 (37.1)	1704 (96.1)	222 (12.5)
	41-60	1601 (33.4)	1521 (95.0)	329 (20.5)
	>60	814 (17.0)	733 (90.0)	193 (23.7)
**Gender**			*P*=.06	*P*=.25
	Male	2248 (47.0)	2108 (93.8)	365 (16.2)
	Female	2540 (53.0)	2413 (95.0)	444 (17.5)
**Marital status**			*P*<.001	*P*<.001
	Unmarried	1725 (36.0)	1654 (95.9)	189 (11.0)
	Married or remarried	2851 (59.5)	2682 (94.1)	566 (19.9)
	Divorced or widowed, not remarried	212 (4.4)	185 (87.3)	54 (25.5)
**Occupation**			*P*<.001	*P*<.001
	Seeking employment	300 (6.3)	274 (91.3)	62 (20.7)
	Not working (not able to work)	273 (5.7)	239 (87.5)	65 (23.8)
	Self-employed shop owner or entrepreneur	569 (11.9)	533 (93.7)	118 (20.7)
	Staff member in a government or public institution	615 (12.8)	599 (97.4)	110 (19.7)
	Farmer, fisherman, or herdsman	321 (6.7)	287 (89.4)	83 (25.9)
	Retired	499 (10.4)	469 (94.0)	112 (22.4)
	Student	1155 (24.1)	1109 (96.0)	102 (8.8)
	Staff member in a big company	276 (5.8)	264 (95.7)	45 (16.3)
	Staff member in a small or medium company	426 (8.9)	410 (96.2)	58 (13.6)
	Other	354 (7.4)	337 (95.2)	54 (15.3)
**Education (years)**			*P*<.001	*P*<.001
	≤6	698 (14.6)	621 (89.0)	160 (22.9)
	7-9	809 (16.9)	758 (93.7)	166 (20.5)
	10-12	865 (18.1)	811 (93.8)	178 (20.6)
	13-16	2145 (44.8)	2064 (96.2)	286 (13.3)
	>16	271 (5.7)	267 (98.5)	19 (7.0)
**Areas**			*P*=.09	*P*=.88
	Eastern China	1317 (27.5)	1259 (95.6)	227 (17.2)
	Central China	2191 (45.8)	2058 (93.9)	371 (16.9)
	Western China	1280 (26.7)	1204 (94.1)	211 (16.5)
**Type of area**			*P*<.001	*P*=.93
	Urban	3065 (64.0)	2928 (95.5)	519 (16.9)
	Rural	1723 (36.0)	1593 (92.5)	290 (16.8)
**Household composition**			*P*=.005	*P*=.36
	Living with others	4370 (91.3)	4139 (94.7)	745 (17.0)
	Living alone	418 (8.7)	382 (91.4)	64 (15.3)
**Relative self-reported individual income**			*P*<.001	*P*=.03
	Low (0%-20%)	1505 (31.4)	1393 (92.6)	266 (17.7)
	Low and middle (20%-40%)	1141 (23.8)	1069 (93.7)	214 (18.8)
	Average (40%-60%)	1816 (37.9)	1743 (96.0)	269 (14.8)
	Upper middle (60%-80%)	285 (6.0)	277 (97.2)	55 (19.3)
	High (80%-100%)	41 (0.9)	39 (95.1)	5 (12.2)
**Household income in 2019, RMB (US $)**			*P*<.001	*P*=.008
	<100,000 (<14,954)	2074 (43.3)	1920 (92.6)	395 (19.0)
	100,000-200,000 (14,954-29,909)	1735 (36.2)	1667 (96.1)	270 (15.6)
	200,000-300,000 (29,909-44,864)	579 (12.1)	556 (96.0)	89 (15.4)
	300,000-400,000 (44,864-59,819)	193 (4.0)	182 (94.3)	23 (11.9)
	>400,000 (59,819)	207 (4.3)	196 (94.7)	32 (15.5)

### Health Perception and Attitude Toward COVID-19

The average health perception score was 4.63 (SD 0.67). The number of perception scores above the average level significantly differed across age groups, categories of marital status, occupational categories, education levels, residence area type, individual income level, and household income in 2019 (*P*<.001). In contrast, significant differences in “misled” rates were only found for age groups, categories of marital status, occupational categories, and education levels (*P*<.001; Table 1).

The overall accuracy per question is shown in Table 2. This study showed that the sample residents had a high level of knowledge of preventive measures against COVID-19, but the accuracy of responses to “Eating a lot of garlic could help to prevent COVID-19” and “If infected, older adults have the highest risk of mortality” was low in comparison to the other questions, suggesting that further education efforts are needed to improve residents' ability to identify rumors and their understanding of the susceptibility of specific groups.

**Table 2:** Questionnaire to examine participants' level of knowledge of COVID-19.

Knowledge	Accuracy (%)
1. Washing your hands and wearing a mask frequently could help to prevent COVID-19.	98.3
2. When people with COVID-19 sneeze or cough around you, is it easier to be infected?	97.0
3. Eating a lot of garlic could help to prevent COVID-19.	83.1
4. Improving your immunity could help you fight COVID-19.	95.5
5. If infected, older adults have the highest risk of mortality.	88.8

**Figure 1:**
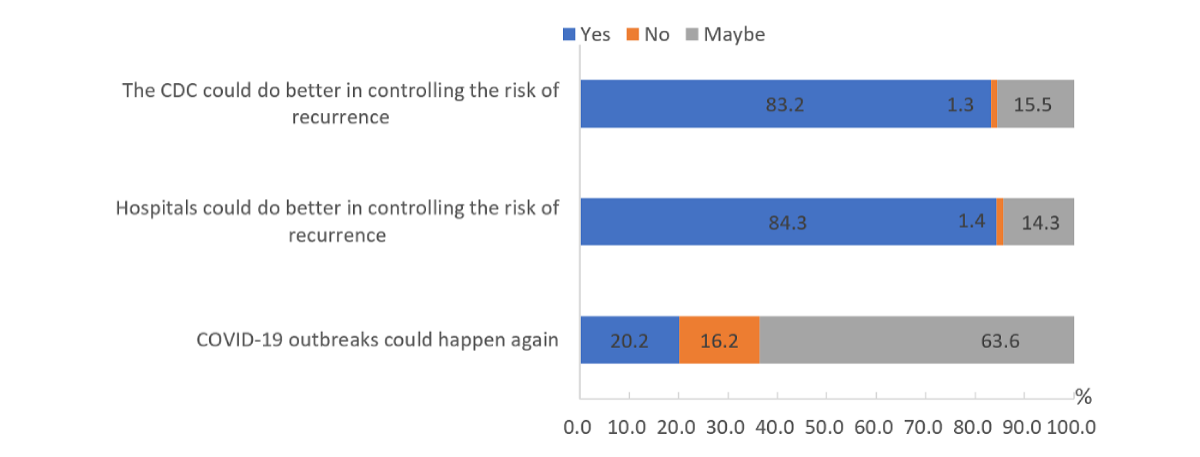
Participants' level of confidence in the success of the fight against the COVID-19 epidemic.

The vast majority of the participants held an optimistic attitude toward the COVID-19 epidemic. In total, 83.2% of them believed that the Centers for Disease Control could do better in controlling the risk of recurrence, 84.3% thought that hospitals could do better in controlling the risk of recurrence, and only 20.2% thought that COVID-19 outbreaks could happen again (Figure 1), which is consistent with the high level of awareness of COVID-19 prevention measures among the participants. Accurate guidance from the Chinese government during the epidemic period enabled Chinese citizens to have a good understanding of COVID-19 and avoid unnecessary panic and confusion, which also increased people's confidence in the success of the fight against the epidemic [22].

### Effect of Various COVID-19 Information Sources

Residents received COVID-19 information through a variety of channels, among which the majority of residents chose mainstream media such as suggestions from experts (76.1%), television (60.0%), and newspapers (57.9%). In addition, it is worth noting that with the advent of the Web 2.0 era and the rise of social media, opinions (46.6%) and videos (42.9%) on social media have also played an important role in this information dissemination. The chi-square test results of answers to the question “Eating a lot of garlic could prevent COVID-19” showed that whether participants obtained information from experts and newspapers is significantly related to whether residents were misled; in particular, expert opinions play a decisive role. During this outbreak, Chinese experts in relevant fields have contributed to refuting rumors by spreading accurate information about COVID-19 through television, the internet, newspapers, and other channels (Table 3).

**Table 3:** Chi-square test results of answers to the question “Eating a lot of garlic could prevent COVID-19.”

Sources of COVID-19 information	Total participants, n	Participants who responded “yes,” % (95% CI)	Participants who responded “no,” % (95% CI)	*χ*^2^ value	*P* value
Phone message or call	2206	44.10 (40.70-47.60)	46.50 (44.90-48.00)	1.48	.22
Suggestions from experts	3642	67.20 (64.00-70.40)	77.90 (76.50-79.10)	41.61	<.001
Video on social media	2056	46.00 (42.60-49.40)	42.30 (40.80-43.90)	3.68	.06
Opinions on social media	2230	45.90 (42.40-49.30)	46.70 (45.20-48.30)	0.20	.65
Newspapers	2771	50.90 (47.50-54.40)	59.30 (57.80-60.80)	19.27	<.001
Television	2871	55.70 (52.30-59.10)	60.80 (59.30-62.30)	7.20	.007
Friends	1238	27.80 (24.80-31.00)	25.50 (24.10-26.80)	1.94	.16
Patient's experience	405	10.50 (8.50-12.80)	8.00 (7.20-8.90)	5.27	.02
Family	1870	42.00 (38.70-45.50)	38.50 (36.90-40.00)	3.61	.06
Own COVID-19 experience	358	9.00 (7.20-11.10)	7.20 (6.40-8.00)	3.37	.07

### Groups That Are Relatively Vulnerable to COVID-19 Misinformation

The results showed that residents from Central China (versus Eastern China, β=–0.342) and those who were divorced or widowed but not remarried (versus unmarried, β=–0.966) were significantly associated with a lower level of basic COVID-19 preventive perception (Table 4). In contrast, those aged 41-60 years (versus <20 years, β=0.9261), staff members in a government or public institution (versus seeking employment, β=0.821), education of >16 years (versus <6 years, β=1.294), and getting information about COVID-19 on television (β=0.683) were associated with higher scores (Table 4).

On the issue of identifying misleading information, students (versus those seeking employment, β=0.791), those with an education of >16 years (versus <6 years, β=0.774), those with one suspected symptom (versus those without suspected symptoms, β=0.494, *P*<.001), and those who take expert advice (β=0.322) were significantly associated with a higher ability to identify misleading information (Table 4). On the contrary, participants with >2 other diseases (versus those without other diseases, β=–0.601) and those with a tendency to get information from videos on social media (β=–0.589) were more likely to be indistinguishable from correct or incorrect information about COVID-19 (Table 4).

**Table 4:** Perception level of COVID-19 by demographic variable and knowledge source.

Variables		Basic COVID-19 preventive perception		Identified the misleading information	
		B	OR^a^ (95% CI)	B	OR (95% CI)
**Areas (reference: Eastern China)**					
	Central China	–0.342^b^	0.71 (0.51-1.00)	0.083	1.09 (0.89-1.32)
	Western China	–0.200	0.82 (0.56-1.20)	0.071	1.07 (0.86-1.34)
Living in a rural area (reference: urban area)		–0.228	0.80 (0.59-1.09)	0.240^b^	1.27 (1.05-1.54)
Living alone (reference: living with others)		–0.373	0.69 (0.45-1.06)	0.202	1.22 (0.90-1.67)
**Age, years (reference: ≤20)**					
	21-40	0.651^b^	1.92 (1.06-3.48)	0.121	1.13 (0.76-1.67)
	41-60	0.926^c^	2.53 (1.25-5.12)	–0.009	0.99 (0.63-1.56)
	>60	0.203	1.23 (0.55-2.74)	–0.041	0.96 (0.56-1.63)
Female (reference: male)		0.214	1.24 (0.95-1.61)	–0.174^b^	0.84 (0.72-0.99)
**Marital status (reference: unmarried)**					
	Married or remarried	–0.574^b^	0.56 (0.32-0.98)	0.001	1.00 (0.72-1.39)
	Divorced or widowed but not remarried	–0.966^c^	0.38 (0.18-0.79)	–0.119	0.89 (0.55-1.42)
**Occupation (reference: seeking employment)**					
	Not working (not able to work)	0.205	1.23 (0.63-2.40)	0.090	1.09 (0.69-1.73)
	Self-employed	0.260	1.30 (0.72-2.32)	0.050	1.05 (0.72-1.52)
	Staff member in a government or public institution	0.821^b^	2.27 (1.11-4.65)	–0.061	0.94 (0.64-1.39)
	Farmer, fisherman, or herdsman	0.138	1.15 (0.61-2.16)	–0.160	0.85 (0.56-1.31)
	Retired	0.691	2.00 (0.99-4.05)	0.127	1.14 (0.73-1.76)
	Student	0.639	1.90 (0.92-3.90)	0.79^c^	2.21 (1.41-3.46)
	Staff member in a big company	0.365	1.44 (0.67-3.12)	0.118	1.13 (0.71-1.78)
	Staff member in a small or medium company	0.604	1.83 (0.92-3.63)	0.387	1.47 (0.98-2.22)
	Other	0.640	1.90 (0.98-3.68)	0.402	1.50 (0.99-2.26)
**Household income in 2019, RMB (reference: <100,000 RMB)**					
	100,000-200,000	0.397^b^	1.49 (1.08-2.05)	0.274^b^	1.31 (1.09-1.59)
	200,000-300,000	0.228	1.26 (0.77-2.05)	0.247	1.28 (0.97-1.69)
	300,000-400,000	–0.21	0.81 (0.41-1.62)	0.466	1.59 (0.99-2.57)
	>400,000	–0.082	0.92 (0.46-1.84)	0.208	1.23 (0.81-1.88)
**Education, years (reference: ≤6 years)**					
	7-9	0.326	1.39 (0.91-2.10)	–0.038	0.96 (0.73-1.26)
	10-12	0.208	1.23 (0.79-1.92)	–0.146	0.86 (0.65-1.15)
	13-16	0.38	1.46 (0.89-2.40)	0.117	1.12 (0.82-1.54)
	>16	1.294^b^	3.65 (1.17-11.34)	0.774^c^	2.17 (1.21-3.89)
**Number of other diseases (reference: 0)**					
	1	0.625^c^	1.87 (1.21-2.88)	–0.040	0.96 (0.76-1.21)
	2	0.145	1.16 (0.67-2.00)	–0.239	0.79 (0.57-1.09)
	>2	0.079	1.08 (0.64-1.84)	–0.601^c^	0.55 (0.39-0.77)
**Number of suspected symptoms (reference: 0)**					
	1	–0.097	0.91 (0.55-1.51)	0.494^c^	1.64 (1.17-2.30)
	2	0.486	1.63 (0.77-3.45)	0.288	1.33 (0.91-1.96)
	>2	–0.294	0.75 (0.43-1.30)	–0.057	0.95 (0.66-1.35)
**Number of COVID-19 information sources (reference: 0)**					
	1	0.750^b^	2.12 (1.14-3.93)	0.443^b^	1.56 (1.07-2.27)
	2	0.396	1.49 (0.86-2.56)	0.219	1.25 (0.87-1.78)
	3	0.695^b^	2.01 (1.10-3.65)	0.294	1.34 (0.92-1.95)
	4	0.911^c^	2.49 (1.38-4.50)	0.434^b^	1.54 (1.05-2.26)
	5	0.795^c^	2.25 (1.19-4.14)	0.911^c^	2.49 (1.59-3.90)
	6	1.074^c^	2.93 (1.47-5.81)	0.764^c^	2.15 (1.36-3.40)
	7	0.993^c^	2.70 (1.30-5.59)	0.433	1.54 (0.94-2.54)
	8	0.804^b^	2.24 (1.07-4.68)	0.297	1.35 (0.79-2.30)
	9	1.449^c^	4.26 (1.41-12.88)	0.662	1.94 (0.95-3.95)
**Knowledge source**					
	Phone message or call	0.346	1.41 (0.94-2.13)	0.037	1.04 (0.82-1.31)
	Suggestions from experts	0.28	1.32 (0.84-2.09)	0.322^b^	1.38 (1.03-1.84)
	Video on social media	–0.404	0.67 (0.40-1.11)	–0.589^c^	0.55 (0.41-0.74)
	Opinions on social media	–0.252	0.78 (0.47-1.30)	–0.038	0.96 (0.72-1.29)
	Newspapers	-0.156	0.86 (0.54-1.36)	0.192	1.21 (0.93-1.58)
	Television	0.683^c^	1.98 (1.31-2.99)	–0.056	0.95 (0.74-1.21)
	Friends	–0.169	0.85 (0.54-1.33)	0.095	1.10 (0.84-1.43)
	Patient's experience	–0.45	0.64 (0.34-1.19)	–0.248	0.78 (0.53-1.16)
	Family	–0.217	0.81 (0.55-1.18)	–0.073	0.93 (0.74-1.16)
	Own COVID-19 experience	–0.522	0.59 (0.32-1.12)	0.165	1.18 (0.77-1.82)
Constant		1.108	3.03	0.874	2.40

^a^OR: odds ratio.

^b^*P*<.05.

^c^*P*<.001.

**Figure 2:**
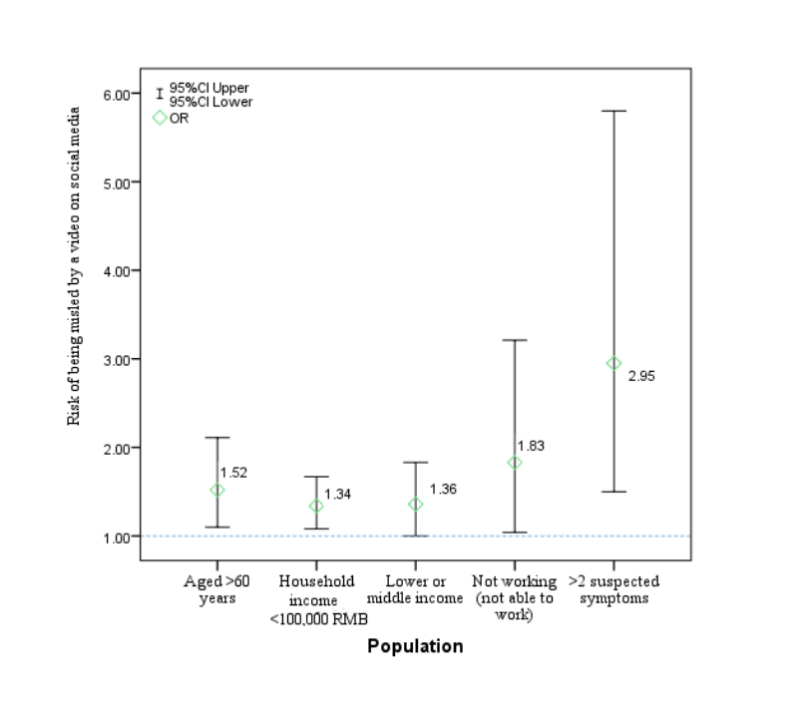
Vulnerable populations and their risk of being misled by incorrect information in videos on social media. OR: odds ratio.

Interestingly, the higher the number of information sources used by a participant, the higher their preventive perception and ability to identify misleading information. People who obtained information from >3 channels showed a significant improvement in their knowledge level compared to those who obtained information from only 1 or 2 channels (n=4, OR 2.49, 95% CI 1.38-4.50; n=9, OR 4.26, 95% CI 1.41-12.88). Videos on social media were the main culprit for the spreading of rumors, while expert advice helped to refute rumors, which is consistent with the results of the chi-square test above. The results of the stratified analysis showed that the population aged >60 years (OR 1.52, 95% CI 1.10-2.11), those with a lower- or middle-income level (OR 1.36, 95% CI 1.00-1.83), those who were not working and not able to work (OR 1.83, 95% CI 1.04-3.21), those with a household income <100,000 RMB (<US $14,954; OR 1.34, 95% CI 1.08-1.67), and those with >2 suspected symptoms (OR 2.95, 95% CI 1.50-5.80) were more likely to be misled by videos on social media (Figure 2). Although expert advice was the most useful tool to dispel rumors, its impact on vulnerable groups such as residents aged >60 years (OR 1.14, 95% CI 0.79-1.64), those who were divorced or widowed but not remarried (OR 0.54, 95% CI 0.25-1.16), those without work (OR 0.91, 95% CI 0.49-1.67), and those with suspected symptoms (OR 0.93, 95% CI 0.41-2.09) was limited (Table 5).

**Table 5:** Hierarchical analysis of the expert suggestion error correction effect among subgroups.

Characteristics		Participants who did not access expert advice	Participants who accessed expert advice	Odds ratio (95% CI)
		Participants who discerned rumors/total number of participants (%)	Participants who did not discern rumors/total number of participants (%)	
**Age (years)**				
	≤20	115/135 (85.2)	419/464 (90.3)	1.62 (0.92-2.85)
	21-40	277/358 (77.4)	1275/1416 (90.0)	2.64 (1.95-3.58)
	41-60	331/441 (75.1)	941/1160 (81.1)	1.43 (1.10-1.85)
	>60	158/212 (74.5)	463/602 (76.9)	1.14 (0.79-1.64)
**Gender**				
	Male	450/570 (78.9)	1433/1678 (85.4)	1.56 (1.22-1.99)
	Female	431/576 (74.8)	1665/1964 (84.8)	1.59 (1.36-1.86)
**Marital status**				
	Unmarried	288/355 (81.1)	1248/1370 (91.1)	2.38 (1.72-3.29)
	Married or remarried	546/734 (74.4)	1739/2117 (82.1)	1.58 (1.30-1.93)
	Divorced or widowed but not remarried	47/57 (82.5)	111/155 (71.6)	0.54 (0.25-1.16)
**Occupation**				
	Seeking employment	52/71 (73.2)	186/229 (81.2)	1.58 (0.85-2.94)
	Not working (not able to work)	65/84 (77.4)	143/189 (75.7)	0.91 (0.49-1.67)
	Self-employed shop owner or entrepreneur	128/172 (74.4)	323/397 (81.3)	1.50 (0.98-2.30)
	Staff member in a government or public institution	107/141 (75.9)	398/474 (84.0)	1.66 (1.05-2.63)
	Famer, fisherman, or herdsman	65/88 (73.9)	173/233 (74.2)	1.02 (0.58-1.78)
	Retired	86/117 (73.5)	301/382 (78.8)	1.34 (0.83-2.16)
	Student	184/220 (83.6)	869/935 (92.9)	2.58 (1.67-3.98)
	Staff member in a big company	55/74 (74.3)	176/202 (87.1)	2.34 (1.20-4.55)
	Staff member in a small or medium company	66/83 (79.5)	302/343 (88.0)	1.90 (1.02-3.54)
	Other	73/96 (76.0)	227/258 (88.0)	2.31 (1.27-4.21)
**Education (years)**				
	≤6	160/207 (77.3)	378/491 (77.0)	0.98 (0.67-1.45)
	7-9	169/228 (74.1)	474/581 (81.6)	1.55 (1.08-2.22)
	10-12	174/234 (74.4)	513/631 (81.3)	1.50 (1.05-2.14)
	13-16	340/434 (78.3)	1519/1711 (88.8)	2.19 (1.66-2.88)
	>16	38/43 (88.4)	214/228 (93.9)	2.01 (0.69-5.91)
**Areas**				
	Eastern China	260/342 (76.0)	830/975 (85.1)	1.81 (1.33-2.45)
	Central China	387/502 (77.1)	1433/1689 (84.8)	1.66 (1.30-2.13)
	Western China	234/302 (77.5)	835/978 (85.4)	1.70 (1.23-2.34)
**Type of area**				
	Urban	546/717 (76.2)	2000/2348 (85.2)	1.80 (1.47-2.21)
	Rural	335/429 (78.1)	1098/1294 (84.9)	1.57 (1.19-2.07)
**Household composition**				
	Living with others	782/1023 (76.4)	2843/3347 (84.9)	1.74 (1.46-2.07)
	Living alone	99/123 (80.5)	255/295 (86.4)	1.55 (0.89-2.70)
**Household income in 2019, RMB (US $)**				
	<100,000 (14,954)	406/524 (77.5)	1273/1550 (82.1)	1.34 (1.05-1.70)
	100,000-200,000 (14,954-29,909)	323/417 (77.5)	1142/1318 (86.6)	1.89 (1.43-2.50)
	200,000-300,000 (29,909-44,864)	100/132 (75.8)	390/447 (87.2)	2.19 (1.35-3.56)
	300,000-400,000 (44,864-59,819)	17/26 (65.4)	153/167 (91.6)	5.79 (2.18-15.35)
	>400,000 (>59,819)	35/47 (74.5)	140/160 (87.5)	2.40 (1.07-5.37)
**Relative self-reported individual income**				
	Low (0%-20%)	287/373 (76.9)	952/1132 (84.1)	1.59 (1.19-2.12)
	20%-40%	216/285 (75.8)	711/856 (83.1)	1.57 (1.13-2.17)
	Average (40%-60%)	333/423 (78.7)	1214/1393 (87.2)	1.83 (1.38-2.43)
	60%-80%	41/59 (69.5)	189/226 (83.6)	2.24 (1.16-4.33)
	High (80%-100%)	4/6 (66.7)	32/35 (91.4)	5.33 (0.67-42.23)
**Number of suspected symptoms**				
	0	745/981 (75.9)	2485/2933 (84.7)	1.76 (1.47-2.10)
	1	67/75 (89.3)	272/307 (88.6)	0.93 (0.41-2.09)
	2	39/48 (81.3)	178/204 (87.3)	1.58 (0.69-3.64)
	>2	30/42 (71.4)	163/198 (82.3)	1.86 (0.87-3.99)
**Number of other diseases**				
	0	662/843 (78.5)	2286/2626 (87.1)	1.84 (1.51-2.25)
	1	137/172 (79.7)	487/597 (81.6)	1.13 (0.74-1.73)
	2	44/64 (68.8)	187/232 (80.6)	1.89 (1.02-3.51)
	>2	38/67 (56.7)	138/187 (73.8)	2.15 (1.20-3.85)

## Discussion

### Principal Findings

Overall, this study indicated a high level of awareness of COVID-19 among Chinese residents; this seems to be primarily related to education and the information sources used. More than half of the study sample had a bachelor’s degree or higher. Our sample had a large number of students (24.1%) and staff in government or public institutions (12.8%), who have a high information acquisition ability, which also contributed to the high accuracy observed in this research. These results are similar to the results of an earlier study in China, in which participants of higher socioeconomic status were more knowledgeable and better able to take appropriate measures to prevent the spread of COVID-19 [22].

The ability to seek information from multiple channels due to the development of the information age had a significant impact. The higher the number of information sources, the more opportunity there is for individuals to acquire knowledge and consolidate existing knowledge; it is also likelier that individuals will be able to effectively discern misinformation from facts. Due to the seriousness of the epidemic, people actively sought knowledge regarding the virus from various information sources, such as CCTV (China Central Television), the official website of the National Health Commission of China, and the official WeChat account of the Wuhan Health Commission [22]. For example, the “news 1+1” column takes the form of a dialogue between a host and an expert to answer the questions about the epidemic that people are most concerned about every day; it played a large role in the dissemination of knowledge and the refutation of rumors. Accurate information provided by trusted clinicians and scientists can help mitigate the spread of misinformation that is damaging to public health. As experts' suggestions played a role in correcting the “eating a lot of garlic could prevent COVID-19” rumor in our sample, health communication specialists may be able to directly counter prominent false narratives while promoting reliable sources of health information [26].

With the development of information technology, social media has played an important role in spreading information, including during and about this outbreak (46.6% and 42.9% of participants got information from opinions and videos on social media, respectively). Although these platforms provide easier and accessible ways of getting or generating information, they can also be a source of misinformation [27]. Fake news on Weibo, WeChat, TikTok, and other short video platforms regarding potential drugs for COVID-19 (including ShuangHuangLian, garlic, and radix) resulted in unnecessary confusion and a shortage of drugs for patients who need them. Uncertainty breeds rumors and confusion, and social media platforms offer a fertile space for misinformation to be generated and disseminated [28]. This study highlighted the need for public health bodies to continue social media campaigns to minimize the circulation of inaccurate information about COVID-19 [23,29].

Among our participants, health perception and the ability to detect rumors was significantly lower among some vulnerable populations, including older participants and those with lower educational and income levels. These people may find it difficult to distinguish truth from misinformation in the news [30]. Meanwhile, they may be influenced by the traditional thinking pattern of “prefer to believe what they have rather than believe what they have not,” which makes them more likely to be misled by rumors and to take preventive measures that do not have any effect. To make matters worse, although we found that listening to expert advice resulted in a higher ability to identify misleading information, expert advice plays a limited role in these vulnerable populations. Therefore, we conclude that health education interventions would be more effective if they focused on the targeted demographic groups [31]; for example, COVID-19 knowledge may be greatly increased if health education programs are specifically designed for older people and persons with a low level of education.

In this study, misleading questions were used to determine the main communication channels of misinformation and the groups that were most misled, which is more targeted than previous studies on knowledge level. However, this study still has some limitations. Rapid online surveys are a promising method to assess and track knowledge and perceptions in the midst of rapidly evolving infectious disease outbreaks [23]. An advantage of this study is that it used a rapid online survey to collect a large number of samples. However, a large proportion of participants were well-educated and engaged in mental work, resulting in a certain bias in the results. For this study, we recruited and trained the responsible investigators located in the sampled cities, who selected the sample by directly sending the questionnaire to families in their social network, which ensured we captured older adults in our sample. For those without the ability to use the internet, it is difficult to control the response process.

### Conclusions

In general, our findings indicate that participants in the survey had a high level of health awareness of COVID-19 and were optimistic about success in the fight against the epidemic, which is important to limit the spread of the disease. Health information is spread through both traditional and new platforms, including television, newspapers, the internet, social media, and short video platforms, and the number of channels used to obtain information was positively associated with health perception. Among information sources, suggestions from experts are the most accurate source, while social media plays a large role in spreading rumors. We found that health knowledge was lower among older adults, those with less education, those who are unemployed or have a lower income, and those with underlying diseases. People in these groups were more likely to be misled by misinformation in videos on social media, while the error correction effect of experts was very limited in vulnerable populations. Although the government has taken major steps to limit the spread of the disease, more effort is needed to strengthen surveillance of social media, implement targeted support, and increase the influence of experts on vulnerable populations to reduce the spreading of rumors.

## Abbreviations

KAP: Knowledge-Attitude-Belief
WHO: World Health Organization
