# Correction: Health Perceptions and Misconceptions Regarding COVID-19 in China: Online Survey Study

**DOI:** 10.2196/39493

**Published:** 2022-07-13

**Authors:** Jiawei Zhou, Bishwajit Ghose, Ruoxi Wang, Ruijun Wu, Zhifei Li, Rui Huang, Da Feng, Zhanchun Feng, Shangfeng Tang

**Affiliations:** 1 School of Medicine and Health Management Tongji Medical College Huazhong University of Science and Technology Wuhan China; 2 School of Pharmacy Tongji Medical College Huazhong University of Science and Technology Wuhan China; 3 China National Center for Biotechnology Development Beijng China

In “Health Perceptions and Misconceptions Regarding COVID-19 in China: Online Survey Study” (J Med Internet Res 2020;22(11):e21099), the authors made six updates.

1. The originally published article indicated the selected provinces in Eastern, Central, and Western China in a map in Figure 1. As this information has already been explained in the text, the figure was deemed unnecessary and was removed in the corrected version of the article. Accordingly, the numbering and in-text citations of the remaining figures were also updated in the corrected article.

2. The original Figure 1's in-text citation appeared in the article as follows:

As seen in Figure 1, Hubei, Hunan, and Shanxi provinces were selected in Central China. In Eastern China, Guangdong, Zhejiang, and Fujian provinces were selected.

In the corrected version, it has been changed to the following:

Hubei, Hunan, and Shanxi provinces were selected in Central China. In Eastern China, Guangdong, Zhejiang, and Fujian provinces were selected.

3. The originally published Figure 2 (captioned “Participants' level of confidence in the success of the fight against the COVID-19 epidemic”) is now renumbered as Figure 1.

4. The original Figure 2's in-text citation appeared in the article as follows:

In total, 83.2% of them believed that the Centers for Disease Control could do better in controlling the risk of recurrence, 84.3% thought that hospitals could do better in controlling the risk of recurrence, and only 20.2% thought that COVID-19 outbreaks could happen again (Figure 2), which is consistent with the high level of awareness of COVID-19 prevention measures among the participants.

In the corrected version, it has been changed to the following:

In total, 83.2% of them believed that the Centers for Disease Control could do better in controlling the risk of recurrence, 84.3% thought that hospitals could do better in controlling the risk of recurrence, and only 20.2% thought that COVID-19 outbreaks could happen again (Figure 1), which is consistent with the high level of awareness of COVID-19 prevention measures among the participants.

5. The originally published Figure 3 (captioned “Vulnerable populations and their risk of being misled by incorrect information in videos on social media. OR: odds ratio”) is now renumbered as Figure 2.

6. The original Figure 3's in-text citation appeared in the article as follows:

The results of the stratified analysis showed that the population aged >60 years (OR 1.52, 95% CI 1.10-2.11), those with a lower- or middle-income level (OR 1.36, 95% CI 1.00-1.83), those who were not working and not able to work (OR 1.83, 95% CI 1.04-3.21), those with a household income <100,000 RMB (<US $14,954; OR 1.34, 95% CI 1.08-1.67), and those with >2 suspected symptoms (OR 2.95, 95% CI 1.50-5.80) were more likely to be misled by videos on social media (Figure 3).

In the corrected version, it has been changed to the following:

The results of the stratified analysis showed that the population aged >60 years (OR 1.52, 95% CI 1.10-2.11), those with a lower- or middle-income level (OR 1.36, 95% CI 1.00-1.83), those who were not working and not able to work (OR 1.83, 95% CI 1.04-3.21), those with a household income <100,000 RMB (<US $14,954; OR 1.34, 95% CI 1.08-1.67), and those with >2 suspected symptoms (OR 2.95, 95% CI 1.50-5.80) were more likely to be misled by videos on social media (Figure 2).

The correction will appear in the online version of the paper on the JMIR Publications website on July 13, 2022, together with the publication of this correction notice. Because this was made after submission to PubMed, PubMed Central, and other full-text repositories, the corrected article has also been resubmitted to those repositories.

